# Sensibilidad *in vitro* a benznidazol, nifurtimox y posaconazol de cepas de *Trypanosoma cruzi* de Paraguay

**DOI:** 10.7705/biomedica.5187

**Published:** 2020-11-12

**Authors:** Nidia Acosta, Gloria Yaluff, Elsa López, Christopher Bobadilla, Analía Ramírez, Ivana Fernández, Patricia Escobar

**Affiliations:** 1 Departamento de Medicina Tropical, Instituto de Investigaciones en Ciencias de la Salud, Universidad Nacional de Asunción, Asunción, Paraguay Universidad Nacional de Asunción Departamento de Medicina Tropical Instituto de Investigaciones en Ciencias de la Salud Universidad Nacional de Asunción Asunción Paraguay; 2 Centro de Investigaciones en Enfermedades Tropicales-CINTROP, Universidad Industrial de Santander, Bucaramanga, Colombia Universidad Industrial de Santander Centro de Investigaciones en Enfermedades Tropicales-CINTROP Universidad Industrial de Santander Bucaramanga Colombia

**Keywords:** Trypanosoma cruzi, enfermedad de Chagas, nitroimidazoles, nifurtimox, triazoles, Paraguay, Trypanosoma cruzi, Chagas disease, nitroimidazoles, nifurtimox, triazoles, Paraguay

## Abstract

**Introducción.:**

*Trypanosoma cruzi,* agente causal de la enfermedad de Chagas, exhibe una sustancial heterogeneidad fenotípica y genotípica que puede influir en las variaciones epidemiológicas y clínicas de la enfermedad, así como en la sensibilidad a los fármacos utilizados en el tratamiento.

**Objetivo.:**

Evaluar la sensibilidad *in vitro* al benznidazol, el nifurtimox y el posaconazol de 40 cepas clonadas de *T. cruzi* de Paraguay, con distintos genotipos, huéspedes y localidades de origen.

**Materiales y métodos.:**

En su estado epimastigote, los parásitos se incubaron en medio de cultivo LIT *(Liver Infusion Tryptose)* con diferentes concentraciones de cada fármaco en ensayos por triplicado. El grado de sensibilidad se estimó a partir de las concentraciones inhibitorias del 50 y el 90% (IC_50_ e IC_90_) y se obtuvieron los valores promedio y la desviación estándar de cada cepa y fármaco. La significación estadística entre grupos se determinó mediante análisis de varianzas con el test no paramétrico de Wilcoxon/Kruskal-Wallis y valores de p<0,05.

**Resultados.:**

Se observó un amplio rango de respuesta a los fármacos. Se identificaron dos grupos de parásitos (A y B) con diferencias significativas en la sensibilidad al benznidazol (p<0,0001), y tres grupos (A, B, C) en cuanto a la sensibilidad al nifurtimox y el posaconazol (p<0,0001).

**Conclusiones.:**

En general, las cepas fueron más sensibles al nifurtimox que al benznidazol y el posaconazol. Estas diferencias evidencian la heterogeneidad de las poblaciones de *T cruzi* que circulan en Paraguay, lo que debe considerarse en el tratamiento y el seguimiento de las personas afectadas.

## Abstract (en)

**Introduction:**

*Trypanosoma cruzi,* the causative agent of Chagas disease, shows substantial phenotypic and genotypic heterogeneity, which can influence the epidemiological and clinical variations of the disease and the sensitivity to the drugs used in the treatment.

**Objective:**

To assess the in vitro susceptibility to benznidazole, nifurtimox, and posaconazole of 40 cloned strains of *T. cruzi* isolated in Paraguay belonging to different genotypes, hosts, and localities.

**Materials and methods:**

We incubated the parasites in their epimastigote stage in LIT culture medium with different concentrations of each drug in triplicate assays. The degree of susceptibility was estimated by the inhibitory concentrations of 50 and 90% (IC_50_ and IC_90_) to obtain the average values and the standard deviation for each strain and drug. We determined the statistical significance between groups by analysis of variances with the Wilcoxon/Kruskal-Wallis non-parametric test and values of p<0.05.

**Results:**

A wide range of drug response was observed. Two groups of parasites (A and B) were identified as having significant differences in susceptibility to benznidazole (p<0.0001), and three groups (A, B, C) to nifurtimox and posaconazole (p<0.0001).

**Conclusions:**

Overall, the isolates were more susceptible to nifurtimox than benznidazole and posaconazole. Such differences highlight the heterogeneity of *T. cruzi* populations circulating in Paraguay, an aspect to consider in the treatment and follow up of patients.

*Trypanosoma cruzi,* agente causal de la enfermedad de Chagas, exhibe una sustancial heterogeneidad fenotípica y genotípica que podría tener cierto grado de influencia en las variaciones epidemiológicas y clínicas observadas en la enfermedad, así como en la sensibilidad a los fármacos utilizados en el tratamiento.

La enfermedad de Chagas, presente principalmente en Suramérica, Centroamérica y México, se considera la infección parasitaria con la mayor carga socioeconómica en América Latina ^1^ y se estima que entre 6 y 7 millones de personas están actualmente infectadas ^2^. Debido a los crecientes movimientos de población, la enfermedad también se ha extendido a otros continentes, convirtiéndose en un problema emergente para la salud pública global ^3^. La cardiopatía chagásica, su principal manifestación clínica grave, limita la capacidad de los afectados para trabajar y reduce la expectativa de vida, en tanto que su atención médica tiene altos costos, lo que se suma a la carga social que todo esto implica ^2^.

Aunque en los últimos años ha habido grandes progresos en el control de la transmisión vectorial y por transfusión de esta enfermedad, especialmente por medio de iniciativas subregionales (Amazonia, América Central, Pacto Andino, Cono Sur) que han permitido la certificación de la interrupción de la transmisión por los vectores principales en varios países ^2^, aún restan ciertos desafíos, sobre todo en cuanto a la sostenibilidad de las estrategias de control y vigilancia entomológica en países endémicos, la transmisión congénita, y el acceso al tratamiento de las personas ya infectadas y de las que presentan reactivación de la infección por inmunosupresión debida al HIV, enfermedades autoinmunitarias, cáncer o trasplantes ^4^^-^^6^, lo que podría poner en riesgo los logros alcanzados.

En ausencia de una vacuna, el tratamiento farmacológico es el aspecto relevante y crítico en el manejo de la enfermedad de Chagas. Los dos únicos fármacos actualmente disponibles son el benznidazol y el nifurtimox, pero su eficacia es limitada en la fase crónica de la infección, además de tener serios efectos secundarios y requerir largos períodos de tratamiento y seguimiento de los pacientes ^7^^,^^8^.

Asimismo, no hay ensayos de rutina con marcadores "de certeza" de la persistencia del parásito después del tratamiento que evalúen su eficacia, a lo que se suma que en ensayos *in vitro* e *in vivo* se ha evidenciado la existencia de cepas de *T. cruzi* naturalmente resistentes a estos fármacos, que podrían provocar el fracaso terapéutico ^9^^-^^11^. También, se ha observado que la resistencia puede ser inducida por diferentes factores, como la administración de dosis inadecuadas y discontinuas con la consecuente aparición de cepas resistentes ^12^^,^^13^. Los esfuerzos recientes en el desarrollo de fármacos para el tratamiento de la enfermedad se han centrado en antifúngicos como el posaconazol y el ravuconazol, que se mostraban prometedores en los experimentos *in vitro* e *in vivo*^14^^,^^15^, pero que, en los ensayos clínicos en humanos demostraron ser menos efectivos que los dos fármacos tradicionales ^16^^,^^17^.

Actualmente, *T. cruzi* se divide en seis grupos, o unidades taxonómicas discretas, clasificados de Tcl a TcVI ^18^. Todos los grupos pueden ser infectivos para el humano y, hasta la fecha, no se ha podido establecer una clara correlación entre una unidad taxonómica discreta específica y las manifestaciones clínicas de la enfermedad; en parte, esto se debe a que, además de la diversidad del agente causal, las características genéticas del huésped tendrían un papel en la patogenia.

Asimismo, en las infecciones naturales las cepas, al ser multiclonales, por lo general están constituidas por varias poblaciones con diferentes tipos de tropismo tisular que modificarían su comportamiento biológico ^19^^-^^21^. En diversos estudios se ha evidenciado una gran variabilidad genética en las poblaciones de *T. cruzi* en Paraguay y se las ha encontrado infectando distintas clases de huéspedes: humanos, insectos triatominos domiciliarios animales silvestres, especialmente armadillos, marsupiales y, últimamente, primates, así como animales domésticos (perros) ^22^^-^^27^.

En el ciclo doméstico se han identificado los seis genotipos (unidades taxonómicas discretas) con predominio de los grupos Tcll, TcV y TcVI, en tanto que, en el ciclo selvático se han identificado hasta la fecha tres genotipos, Tcll, Tclll y TcV, con predominio del Tclll, todos ellos en animales silvestres. Esta diversidad genética podría tener implicaciones en las manifestaciones clínicas de la enfermedad, la capacidad infecciosa, la virulencia, la patogenia, la capacidad antigénica y la sensibilidad a los medicamentos, aspectos que hasta la fecha se han explorado poco en las cepas paraguayas.

El objetivo del presente estudio fue evaluar la sensibilidad *in vitro* frente a los fármacos tripanocidas de cepas de *T. cruzi* aisladas de diferentes huéspedes en Paraguay, con el fin de conocer si existían diferencias en su sensibilidad e identificar aquellas con resistencia natural.

Esta información permitirá avanzar en el conocimiento de la biología de las poblaciones de *T. cruzi* que circulan en Paraguay, especialmente en cuanto a su grado de sensibilidad a los medicamentos, ya que el éxito del tratamiento es una de las principales estrategias para disminuir la oferta parasitaria y mejorar el pronóstico de la enfermedad de Chagas.

## Materiales y métodos

### Parásitos

Se utilizaron 40 cepas de *T. cruzi* obtenidas entre 1986 y 2013 y pertenecientes a la colección de cepas del Departamento de Medicina Tropical (llCS-UNA), provenientes de humanos, triatominos y animales silvestres de zonas endémicas para la enfermedad de Chagas en Paraguay (cuadro 1). Las cepas se mantenían con pasajes regulares en medio de cultivo y ratón, para luego someterlas de nuevo a criopreservación, con el fin de conservar sus características biológicas. Como cepas de referencia, se emplearon la CL Brener y la Y del lnstituto René Rachou de Belo Horizonte (Brasil), consideradas, respectivamente, como sensible y medianamente resistente al benznidazol y nifurtimox, según Filardi, *et al.*^9^.

**Cuadro 1:** Valores de lC_50_ e lC_90_ obtenidos con los clones de *Trypanosoma cruzi* estudiados en ensayos *in vitro* con tres fármacos tripanocidas. Se muestran los valores promedios de tres pruebas independientes hechas por triplicado ± la desviación estándar.

Cepa	UTD	Huésped	Origen	Benznidazol			Nifurtimox			Posaconazol		
				IC50 (μg/ml) Promedio ± DE	IC90 (μg/ml) Promedio ± DE	Grupo	IC50 (μg/ml) Promedio ± DE	IC90 (μg/ml) Promedio ± DE	Grupo	IC50 (mg/ml) Promedio ± DE	IC90 (mg/ml) Promedio ± DE	Grupo
T601cl1	TcV	*T. i.*	CHA	5,35 ± 2,56	37,44 ± 0,32	A	2,35 ± 0,02	5,81 ± 0,04	A	0,95 ± 0,04	4,75 ± 0,04	A
T632cl1	TcV	*T i.*	CHA	5,01 ± 1,33	36,77 ± 0,51	A	2,13 ± 0,09	6,45 ± 0,02	A	1,34 ± 0,02	4,07± 0,04	A
T720cl1	ND	*T.s.*	CHA	4,23 ± 0,67	42,77 ± 3,31	A	2,34 ± 0,21	5,85 ± 0,18	A	1,19 ± 0,22	3,77 ± 0,16	A
MA125cl1	TclII	*E. s.*	CHA	2,99 ± 0,02	36,21 ± 2,67	A	2,66 ± 0,09	5,96 ± 0,01	A	1,49 ± 0,13	3,66 ± 0,33	A
CON14cl1	TcVI	*T i*	CON	6,01 ± 0,01	37,11 ± 2,75	A	2,61 ± 0,24	6,31 ± 0,04	A	2,33 ± 0,24	4,02 ± 0,22	A
MA26 cl1	Tclll	*D.n.*	CHA	4,25 ± 1,56	35,04 ± 1,10	A	2,77 ± 0,19	6,49 ± 0,17	A	0,45 ± 0,15	3,48 ± 0,29	B
Armadillo20cl2	Tclll	*D.n.*	CHA	4,91 ± 1,61	35,43 ± 0,62	A	1,98 ± 0,01	5,77 ± 0,03	A	0,37 ± 0,12	4,07 ± 0,27	B
CON10cl1	Tcll	*T i.*	CON	6,71 ± 1,70	38,11 ± 0,27	A	1,77 ± 0,22	6,01 ± 0,02	A	0,43 ± 0,2	3,45 ± 0,09	B
RFcl1	TcII	HUM	COR	6,93 ± 2,76	37,90 ± 0,91	A	2,27 ± 0,38	5,93 ± 0,07	A	<0,089	2,32 ± 0,13	C
Pot7bug9cl1	Tcll	*T i.*	CHA	6,58 ± 0,06	41,88 ± 2,75	A	0,80 ± 0,06	5,29 ± 0,01	B	1,49 ± 0,34	4,03 ± 0,06	A
SPE3cl1	ND	*T i.*	SP	5,98 ± 1,07	36,42 ± 0,34	A	0,86 ± 0,10	5,75 ± 0,11	B	1,33 ± 0,03	4,55 ± 0,66	A
CON11cl1	Tcll	*T i*	CON	2,17 ± 0,70	35,82 ± 0,45	A	1,23 ± 0,03	5,66 ± 0,12	B	0,39 ± 0,19	3,98 ± 0,34	B
ARcl1	Tcll	HUM	CEN	8,42 ± 2,66	43,98 ± 2,78	A	1,29 ± 0,40	7,84 ± 0,14	**	0,48 ± 0,16	2,90 ± 0,13	B
Chaco19cl1	Tclll	*T i.*	CHA	5,54 ± 1,41	36,22 ± 1,58	A	1,82 ±0,03	5,54 ± 0,21	B	0,35 ± 0,01	3,25 ± 0,01	B
Armadillo13cl1	Tclll	*D. n.*	CHA	4,33 ± 1,95	42,64 ± 1,88	A	1,31 ± 0,01	5,78 ± 0,06	B	0,68 ± 0,03	2,87 ± 0,30	B
T595cl2	TcV	*T i.*	CHA	7,69 ± 0,45	36,67 ± 0,29	A	1,29 ± 0,01	5,35 ± 0,08	B	<0,089	2,62 ± 0,05	C
MA239cl2	Tclll	*D. n.*	SP	6,60 ± 3,52	32,32 ± 2,87	A	0,93 ± 0,09	6,07± 0,07	B	<0,089	2,17 ± 0,03	C
MA194cl1	Tclll	*Ch. spp*	CHA	4,57 ± 1,08	42,45 ± 2,98	A	0,88 ± 0,22	5,88 ± 0,02	B	3,03 ± 0,17	5,91± 0,10	^**^
JFcl1	TcVl	HUM	AMA	8,50 ± 1,40	94,08 ± 9,24	**	1,84 ± 0,28	5,79 ± 0,12	A	2,09 ± 0,05	4,04 ± 0,86	A
T589cl1	TcV	*T i.*	CHA	14,14 ± 1,01	37,40 ± 1,01	**	2,90 ± 0,50	9,31 ± 0,89	**	3,28 ± 0,03	6,65 ± 0,09	^**^
MANUcl1	ND	HUM	SP	2,68 ± 1,14	34,23 ± 2,77	B	2,28 ± 0,34	6,55 ± 0,02	A	1,85 ± 0,01	4,41 ± 0,23	A
CON3cl1	Tcll	*T.s.*	CON	< 0,625	27,09 ± 1,98	B+	2,18 ± 0,01	6,23 ± 0,05	A	1,24 ± 0,03	4,15 ± 0,03	A
T530cl1	TcV	*T i.*	CHA	3,58 ± 0,23	34,56 ± 1,73	B	1,88 ± 0,07	5,59 ± 0,02	A	0,47 ± 0,11	3,33 ± 0,24	B
T532cl4	TcV	*Ti.*	CHA	2,01 ± 0,23	33,45 ± 2,30	B	2,11 ± 0,06	5,72 ± 0,04	A	0,36 ± 0,21	3,15 ± 0,05	B
MA111cl1	Tclll	*E.s.*	CHA	3,27 ± 1,32	34,13 ± 0,82	B	2,09 ± 0,10	5,77 ± 0,05	A	0,54 ± 0,06	2,72 ± 0,05	B
T503cl1	TcV	*T i.*	CHA	< 0,625	22,96 ± 3,11	B+	2,43 ± 0,32	6,18 ± 0,05	A	<0,089	2,55 ± 0,31	C
CON7cl1	Tcll	*T s.*	CON	< 0,625	26,78 ± 3,01	B+	0,92 ± 0,03	5,18 ± 0,18	B	2,25 ± 0,26	4,89 ± 0,09	A
Pot7bug3cl1	Tcll	*Ti.*	CHA	< 0,625	24,89 ± 1,74	B+	0,82 ± 0,06	5,54 ± 0,03	B	1,93 ± 0,04	4,27 ± 0,08	A
Lengua15cl1	TcVl	*T i.*	CHA	1,71 ± 0,73	35,86 ± 1,13	B	1,10 ± 0,57	5,38 ± 0,09	B	0,32 ± 0,12	3,01 ± 0,15	B
T583cl1	TcV	*Ti.*	CHA	< 0,625	25,45 ± 2,09	B+	0,86 ± 0,22	6,13 ± 0,05	B	<0,089	2,15 ± 0,19	C
MJcl2	ND	HUM	ND	< 0,625	31,16 ± 1,34	B+	<0,156	4,68 ± 0,03	C	1,52 ± 0,002	2,79 ± 0,02	A
CON5cl2	Tcll	*T.s.*	CON	< 0,625	28,44 ± 1,49	B+	<0,156	4,34 ± 0,18	C	<0,089	2,52 ± 0,01	C
MA87cl1	Tclll	*D. n.*	CHA	< 0,625	31,47 ± 1,17	B+	<0,156	5,05 ± 0,07	C	<0,089	2,34 ± 0,06	C
T592cl1	TcV	*Ti.*	CHA	< 0,625	28,81 ± 2,54	B+	<0,156	4,78 ± 0,23	C	<0,089	2,19 ±0,05	C
Armadillo9cl1	Tclll	*D. n.*	CHA	< 0,625	31,19 ± 1,35	B+	<0,156	5,15 ± 0,09	C	<0,089	2,40 ±0,03	C
Chaco32cl2	Tclll	*T i.*	CHA	< 0,625	22,49 ± 2,15	B+	<0,156	5,19 ± 0,04	C	<0,089	2,38 ± 0,22	C
T564cl1	TcV	*T i.*	CHA	< 0,625	23,56 ± 2,27	B+	<0,156	5,25± 0,06	C	<0,089	2,45 ± 0,22	C
T505cl1	TcV	*Ti.*	CHA	< 0,625	25,66 ± 2,23	B+	<0,156	4,76 ± 0,15	C	<0,089	2,49 ± 0,14	C
T591cl1	TcV	*Ti.*	CHA	< 0,625	29,21 ± 2,33	B+	<0,156	5,05 ± 0,08	C	<0,089	2,59 ± 0,05	C
T657cl1	TcV	*T i.*	CHA	< 0,625	28,09 ± 3,54	B+	<0,156	4,98 ± 0,19	C	<0,089	2,44 ± 0,24	C
Y+	Tcll	HUM	BRA	15,22 ± 3,10	43,34 ± 1,70	REF	1,42 ± 0,18	6,35 ± 0,56	REF	2,73 ± 0,05	7,09 ± 0,09	REF
CL Brener	TcVl	*Ti.*	BRA	13,14 ± 0,21	39,47 ± 0,67	REF	1,55± 0,45	5,65± 0,15	REF	6,88 ± 0,17	15,08± 0,38	REF

UTD: unidades taxonómicas discretas, según Zingales, et al. ^18^; HUM: humano; *T.i.: Triatoma infestans: Ts*.: *Triatoma sordida; D.n.: Dasypus novemcinctus; E.s.: Euphractus sexcinctus;* CHA: Chaco; CON: Concepción; SP: San Pedro; COR: Cordillera; AMA: Amambay; BRA: Brasil; REF: cepas de referencia. ND: no determinado. DE: desviación estándar

^**^ Por separado

^+^ En el grupo C por lC_50_

### Mantenimiento de los parásitos

Los parásitos criopreservados a -80°C se recuperaron y se mantuvieron en medio de cultivo LlT *(Liver Infusion Tryptose)*^28^ con suplemento de 10 % de suero fetal bovino a 28 °C; se hicieron pasajes semanales en el medio de cultivo hasta la realización de los ensayos.

### Clonación de los aislamientos

Cada cepa de *T. cruzi* se clonó utilizando la técnica de plaqueo en medio sólido según Yeo, *et al.*^29^, para utilizar un solo clon de cada cepa en todos los ensayos y evitar los posibles efectos de la 'multiclonalidad' característica de este parásito ^20^^,^^21^. Se aislaron cuatro clones de cada cepa utilizando el que presentara el mayor crecimiento en cultivo. Los clones se denominaron con el nombre de la cepa original, seguido de "cl" y el número del respectivo clon, por ejemplo, clon RF cl1.

### Tipificación de los clones biológicos

La extracción de ADN se hizo a partir de un número aproximado de 1 x 10^9^ parásitos por ml provenientes de cultivos de cada clon, utilizando el estuche DNeasy™ (Qiagen), según las instrucciones del fabricante.

Para la tipificación y asignación de las unidades taxonómicas discretas, se utilizó una combinación de cuatro marcadores, incluidos los productos de amplificación de las subunidades pequeña (18S) y grande (24Sα) del gen del ácido ribonucleico ribósomico (ARNr), la región intergénica del miniexón y los perfiles de la reacción en cadena de la polimerasa del gen de la proteína de choque térmico 60 con la enzima de restricción Eco RV *(Hsp* 60/Eco RV-PCR-RFLP), según protocolos ya descritos ^30^^,^^31^.

Los productos amplificados se separaron por electroforesis en geles de agarosa (Sigma) con solución tampón tris-borato-EDTA (TBE) 0,5X teñida con bromuro de etidio y se visualizaron bajo luz ultravioleta. Se utilizaron como referencia las cepas X10 Clone l (Tcl), Esmeraldo-Cl3 (Tcll), ARMA 13 (Tclll), CAN lll (TclV), SC43 (TcV) y CL Brener (TcVl).

### Fármacos

Se usaron benznidazol en comprimidos de 100 mg (Abarax^®^, Laboratorio Farmacéutico Elea, Argentina), nifurtimox en polvo (Sigma-Aldrich) y posaconazol en polvo (Sigma-Aldrich). Se prepararon soluciones madre *(stock)* de cada fármaco, disolviéndolo en dimetilsulfóxido (DMSO), soluciones que luego se diluyeron en diferentes concentraciones con medio de cultivo fresco para las pruebas *in vitro,* cuidando que la concentración del DMSO no excediera el 1 % (v/v) para evitar su efecto citotóxico en los parásitos.

### Ensayos in vitro con los fármacos

El inóculo inicial fue de 5 x 10^5^ parásitos/ml. Los parásitos en estadio epimastigote y fase exponencial de crecimiento, se incubaron en medio de cultivo LlT con diferentes concentraciones de los fármacos en placas de 96 pozos durante 72 horas a 28 °C. Cada prueba se hizo por triplicado en tres eventos independientes. Como controles, se utilizaron parásitos incubados en medio de cultivo sin fármacos, pero con la concentración equivalente de DMSO. La viabilidad de los parásitos se determinó por recuento microscópico en cámara de Neubauer (número de parásitos/ml).

### *Determinación de IC*_*50*_ *y de IC*_*90*_

La actividad tripanocida de los fármacos se estimó con base en los valores de la concentración inhibitoria del 50 % y la del 90 % (lC_50_ e lC_90_, respectivamente). Para ello, se utilizaron los valores obtenidos en el recuento de parásitos y se calcularon estos parámetros mediante un análisis de regresión lineal utilizando el *software* Msxlfit™ (lD Business Solution, Guildford, UK). En cada prueba, se determinaron el promedio y la desviación estándar por cepa y por fármaco.

### Análisis estadísticos

Para determinar la significación estadística entre los valores de lC_50_ e lC_90_ en los grupos, se hizo un análisis de varianzas mediante el test no paramétrico de Wilcoxon/Kruskal-Wallis después de la corrección de Bonferroni y con valores de p<0,05. La prueba de diferencia honestamente significativa *(Honestly Significant Difference,* HSD) de Tukey-Kramer se utilizó para graficar las diferencias significativas entre grupos utilizando el *software* JMP, versión 9.2™ (SAS, Institute).

### Consideraciones éticas

El protocolo fue aprobado por el Comité Científico y el Comité Ético del Instituto de Investigaciones en Ciencias de la Salud de la Universidad Nacional de Asunción (código P25/13).

## Resultados

### Conformación del panel de cepas y clonación

**Figura 1:**
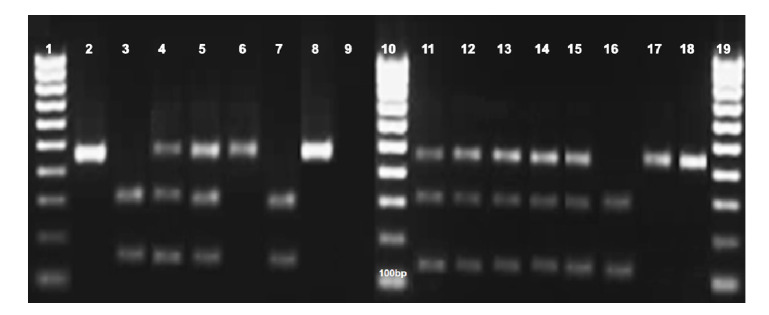
Electroforesis en gel de agarosa de los productos de la PCR-RFLP del gen Hsp60/ *Eco* RV en cepas de *Trypanosoma cruzi.* Líneas 1, 10 y 19: marcadores de peso molecular (100-1.000 bp); 2: cepa de referencia X10 Clone l (Tcl) (462 bp); 3: cepa de referencia ARMA 13 (Tclll) (314/148 bp); 4: cepa de referencia SC43 (TcV) (462/314/148 bp); 5: cepa de referencia CL Brener (TcVl) (462/314/148 bp);6: cepa de referencia Esmeraldo-Cl3 (Tcll) (462 bp);7: clon MA194cl1(Tclll) (314/148 bp); 8: clon CON7cl1 (Tcl o Tcll o TclV) (462 bp); 9:control negativo; 11: clon T595cl2 (TcV o TcVl) (462/314/148 bp); 12: clon T632cl1 (TcV o TcVl) (462/314/148 bp); 13: clon T589cl1 (TcV o TcVl) (462/314/148 bp);14: clon T592cl1 (TcV o TcVl) (462/314/148 bp); 15: clon Lengua15cl1 (TcV o TcVl) (462/314/148 bp); 16: clon MA26cl1(Tclll) (314/148 bp); 17: clon CON10cl1 (Tcl o Tcll o TclV) (462 bp); 18: clon RFcl1 (Tcl o Tcll o TclV) (462 bp)

Todas las cepas de *T. cruzi* se sacaron de criopreservación y se clonaron (cuadro 1). Los clones caracterizados mostraron las bandas esperadas para las correspondientes unidades taxonómicas discretas según estudios previos (30,31). En su mayoría pertenecieron a la unidad taxonómica discreta TcV (32,5 %), seguida por TclII (275 %), TclI (22,5 %) y TcVI (75 %). En cuatro clones (10 %) no fue posible determinar la unidad taxonómica discreta por inconvenientes con las muestras de ADN. En la figura 1 se muestran los productos de la PCR-RFLP del gen *Hsp 60/Eco RV* de un grupo de las cepas analizadas.

En cuanto al tipo de huésped, el 55 % (22/40) se aisló en ejemplares de *Triatoma infestans* intradomiciliarios, el 22,5 % (9/40) de diferentes especies de armadillos capturados en el medio silvestre, el 12,5 % (5/40) de casos clínicos humanos y el 10 % (4/40) de ejemplares domiciliarios de *T. sordida.*

Con respecto a la procedencia, el 67,5 % (27/40) provenía de la región del Chaco, el 15 % (6/40), del Departamento de Concepción, el 7,5 % (3/40), de San Pedro y, una cepa (2,5 %) de Cordillera, otra de Central y otra de Amambay, en tanto que uno de los aislamientos no tenía registro de la procedencia.

### Ensayos in vitro

**Figura 2:**
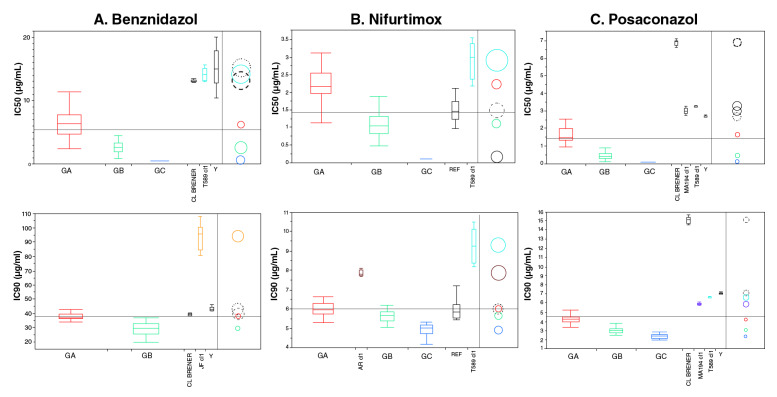
Comparación de los valores de IC_50_ y de IC_90_ de los grupos conformados por cepas de *Trypanosoma cruzi* de Paraguay en ensayos *in vitro* con benznidazol (A), nifurtimox (B) y posaconazol (C). Diagramas en caja de la distribución de los valores de IC50 e IC90 obtenidos en tres pruebas independientes hechas por triplicado con cada una de las cepas según grupo y fármaco. Se indican los percentiles 90 y 10, la mediana y el intervalo de confianza. Los círculos no superpuestos de la derecha indican diferencias significativas (p<0,0001) entre grupos según el test no paramétrico de Kruskal-Wallis y la prueba de Tukey-Kramer (gráfico en el *software* JMP).

GA: grupo A; GB: grupo B; GC: grupo C; REF: cepas de referencia

*Benznidazol.* Los clones analizados fueron sensibles al benznidazol en las concentraciones evaluadas (µg/ml): 50; 37,5; 25; 12,5; 6,25; 2,5; 1,25 y 0,625 (equivalente a un rango de 2,4 a 192 µM), con valores de IC_60_ e IC_90_ variables (cuadro 1). El análisis estadístico de los valores de la IC_60_ permitió clasificarlos en tres grupos (A, B y C), con diferencias significativas en cuanto a la sensibilidad (p<0,0001) (figura 2A). El grupo A, conformado por 18 clones (45 %), fue el menos sensible, con valores de IC_60_ entre 2,17 ± 0,70 µg/ml y 8,42 ± 2,66 µg/mlL. En el grupo B, se clasificaron cinco clones (12,5 %) de sensibilidad intermedia con valores de IC_60_ entre 1,71 ± 0,73 µg/ml y 3,58 ± 0,23 µg/ml, y en el grupo C, 15 clones (37,5 %) con mayor sensibilidad al fármaco e IC_60_ menor de 0,62 µg/ml (la menor concentración evaluada).

Con base en la IC_90_, se determinaron dos grupos, ya que los clones del grupo C quedaron agrupados con los del grupo B. El clon T589cl1, obtenido de un ejemplar intradomiciliario de *T.infestans,* registró el valor más alto de IC_60_ de los clones analizados (14,14 ± 1,01 µg/ml). El máximo porcentaje de inhibición alcanzado con este clon fue del 96 % en promedio. El clon JFcH, aislado de un caso clínico humano, presentó el valor más alto de IC_90_ (94,08 ± 9,24 µg/ml) con una diferencia significativa frente a los clones del grupo A y B y las cepas de referencia (p<0,0001), aunque para la IC_60_ no mostró variación significativa frente a los valores del grupo A. El máximo porcentaje de inhibición alcanzado con este clon fue del 68 %.

*Nifurtimox.* Las concentraciones analizadas fueron de 7,8; 6,25; 3,12; 1,56; 0,625; 0,31 y 0,156 µg/ml (equivalentes a un rango de 0,54 a 27,14 µM) (cuadro 1). Con base en los valores obtenidos de IC_60_ e IC_90_, se determinaron tres grupos (A, B y C) con diferencias significativas en cuanto a la sensibilidad (p<0,0001) (figura 2B). El grupo A fue el menos sensible, con valores de IC_50_ entre 1,77 ± 0,22 µg/ml y 2,77 ± 0,19 µg/ml y de IC_90_ entre 5,72 ± 0,04 µg/ml y 6,55 ± 0,02 µg/ml, e incluyó 16 clones (40 %). El grupo B, integrado por 13 clones (32,5 %), presentó sensibilidad intermedia al fármaco, con valores de IC_50_ entre 0,80 ± 0,06 µg/ml y 1,82 ± 0,03 µg/ml, y de IC_90_, entre 5,18 ± 0,18 µg/ml y 6,13 ± 0,05 µg/ml. El grupo C incluyó 10 clones (25 %) y fue el más sensible, con valores de IC_50_ menores de 0,156 µg/ml (la menor concentración evaluada) y, de IC_90_, entre 4,34 ± 0,18 µg/ml y 5,25 ± 0,06 µg/ml.

El clon T589cl1 registró los valores más altos de IC_50_ e IC_90_ (2,90 ± 0,50 µg/ml y 9,31 ± 0,89 µg/ml, respectivamente). El máximo porcentaje de inhibición alcanzado con este clon fue de 75 %. El clon ARcl1, aislado de un caso clínico humano, presentó valores de IC_90_ (7,84 ± 0,14 µg/ml) significativamente mayores que los de las cepas de referencia. El máximo porcentaje de inhibición alcanzado con este clon fue de 88 %.

*Posaconazol.* La actividad tripanocida de este fármaco se evaluó en un principio utilizando las mismas concentraciones del benznidazol y nifurtimox. Como no se observaba inhibición en el crecimiento de los parásitos, se fue aumentando la dosis y se pasó la concentración de µg a mg. Así, las concentraciones evaluadas fueron de 3,75; 2,68; 1,87; 1,25; 0,93; 0,71; 0,53; 0,35, 0,17 y 0,08 mg/ml (equivalentes a un rango de 126,99 a 5.351,02 µM). Se determinaron tres grupos (A, B y C) con diferencias significativas (p<0,0001) (figura 2C). El grupo A fue el menos sensible, con valores de IC_50_ entre 0,95 ± 0,04 mg/ml y 2,33 ± 0,24 mg/ml y valores de IC_90_ entre 2,79 ± 0,02 mg/ml y 4,89 ± 0,09 mg/ml, e incluyó 13 (32,5 %) cepas (cuadro 1). El grupo B estuvo conformado por 11 clones (27,5 %) con sensibilidad intermedia y valores de IC_50_ entre 0,32 ± 0,12 mg/ml y 0,68 ± 0,03 mg/ml, y de IC_90_, entre 2,72 ± 0,05 mg/ml y 3,98 ± 0,34 mg/ml. El grupo C incluyó 14 clones (35 %) con mayor sensibilidad al fármaco y una IC_50_ menor de 0,089 mg/ml (la menor concentración evaluada), y de IC_90_, entre 2,15 ± 0,19 mg/ml y 2,62 ± 0,05 mg/ml.

El clon T589cl1 registró los valores más altos de IC_50_ y de IC_90_ (3,28 ± 0,03 mg/ml y 6,65 ± 0,09 mg/ml, respectivamente). El máximo porcentaje de inhibición alcanzado con este clon fue, en promedio, de 48 %. El clon MA194cl1, aislado de un armadillo (ciclo selvático), también presentó valores altos de IC_50_ e IC_90_ (3,03 ± 0,17 mg/ml y 5,91 ± 0,10 mg/ml, respectivamente). El máximo porcentaje de inhibición alcanzado con este clon fue de 52 %. Se observó una acentuada diferencia entre las cepas de referencia con este fármaco, siendo la CL Brener menos sensible que la Y.

### Comparación de la sensibilidad de los clones de Trypanosoma cruzi frente a los fármacos

Los clones estudiados fueron más sensibles al nifurtimox que al benznidazol y poco sensibles al posaconazol, ya que fue necesario aumentar la concentración de este último para obtener los valores de las concentraciones inhibitorias en el mismo periodo (72 horas).

Se observó una amplia variación en la sensibilidad a estos fármacos, independientemente de la unidad taxonómica discreta, el huésped y la localidad de origen. Seis clones fueron los de menor sensibilidad a los tres fármacos simultáneamente: T589cl1 (TcV), T601cl1 (TcV), T632cl1 (TcV), T720cl1, MA125cl1 (TcIII) y CON14cl1 (TcVI) (cuadro 1), en tanto que nueve fueron los más sensibles: CON5cl2 (TcII), MA87cl1 (TcIII), T592cl1 (TcV), Armadillo9cl1 (Tclll), Chaco32cl2 (Tclll), T564cl1 (TcV), T505cl1(TcV), T591cl1 (TcV) y T657cl1 (TcV). Los restantes clones mostraron variaciones en su sensibilidad, por ejemplo, el clon MJcl2 presentó sensibilidad intermedia al benznidazol (grupo B), alta al nifurtimox (grupo C) y baja al posaconazol (grupo A).

## Discusión

Entre los factores que pueden influir en la efectividad del tratamiento de la enfermedad de Chagas, están las características genéticas y biológicas de las cepas de *T. cruzi* que predominan en una determinada área geográfica. Por lo tanto, la resistencia natural de algunas cepas a los fármacos tripanocidas podría derivar en bajos porcentajes de cura en los pacientes chagásicos tratados.

En el presente estudio, se investigó la sensibilidad *in vitro* a tres fármacos en 40 cepas de *T. cruzi* de Paraguay. Se determinaron dos poblaciones con diferencias significativas en cuanto a su sensibilidad al benznidazol y tres grupos con respecto al nifurtimox y el posaconazol.

En estudios experimentales en otros países, se ha evidenciado también un amplio rango de respuesta a los fármacos tripanocidas entre las cepas de *T. cruzi.* En sus pruebas en ratones con cepas brasileras, Andrade, *et al.*^32^, observaron diferencias en los porcentajes de cura, con valores menores de 20 %, entre 20 y 50 % y mayores de 50 %. Filardi, *et al.*^9^, también reportaron diferencias en el grado de sensibilidad en cepas de Brasil, y encontraron cepas resistentes al benznidazol y al nifurtimox. En los estudios *in vitro* de Mejía-Jaramillo, *et al.*^11^ con 33 cepas colombianas de *T. cruzi,* se observó que el 36 % fue sensible, el 48 % parcialmente resistente y el 16 % resistente al benznidazol. Luna, *et al.*^10^, en ensayos *in vitro* con cepas de Colombia, y Muñoz-Calderón, *et al.*^33^, con cepas de pacientes en Venezuela, también reportaron diferencias significativas en cuanto a su sensibilidad a los fármacos.

Las razones de estas diferencias entre las cepas de *T. cruzi* todavía no han sido esclarecidas y, hasta la fecha, no se han encontrado marcadores de resistencia natural. Varios autores han propuesto que tales diferencias podrían deberse a cambios en la expresión de importantes proteínas involucradas en la activación de los fármacos, como la enzima mitocondrial nitrorreductasa (NTR) de tipo l dependiente de NADH ^34^^-^^36^, en procesos de desintoxicación, como las enzimas aldoceto reductasa (TcAKR), alcohol deshidrogenasa (TcADHJ ^37^ o prostaglandina F2 alfa sintetasa *(Old Yellow Enzyme,* OYE) ^38^^,^^39^,en el transporte de sustancias a través de la membrana, como la glucoproteína-P (bomba de eflujo) ^40^,y en el metabolismo de las purinas, como la enzima adenina fosforibosiltransferasa (APRT) ^41^, entre otras.

Nosaki, *et al.*^42^, observaron que las cepas de *T. cruzi* con resistencia al nifurtimox presentaban cambios en el ADN, incluido el reordenamiento de cromosomas y la aneuploidía. García-Huertas, *et al.*^41^, identificaron más de 130 genes involucrados en diferentes vías metabólicas que serían regulados por el parásito para resistir al fármaco, y propusieron que la resistencia en *T. cruzi* sería un proceso multigénico. En futuros estudios, se deben evaluar estos aspectos en las cepas analizadas en el presente estudio para dilucidar si tendrían alguna influencia en el grado de sensibilidad.

Las diferencias en la sensibilidad de los clones de Paraguay no parecen relacionadas con las unidades taxonómicas discretas, el tipo de huésped ni la procedencia. En estudios previos, se ha explorado esa posible asociación entre la diversidad filogenética y la sensibilidad a los fármacos. Al evaluar el efecto del benznidazol durante la fases aguda y crónica en la infección experimental en ratones BALB/c, Toledo, *et al.*^43^, encontraron que las cepas de *T. cruzi* I (con la denominación de genotipo 20 en aquel tiempo) eran muy resistentes, en tanto que las cepas de *T. cruzi* II (genotipo 39) fueron parcialmente resistentes y otras cepas de *T cruzi* II (genotipo 32) resultaron sensibles. Sin embargo, en pruebas *in vitro,* Villareal, *et al.*^44^, determinaron la IC_50_ de 16 cepas pertenecientes a diferentes unidades taxonómicas discretas, huéspedes y regiones geográficas, y no encontraron asociación con el grado de sensibilidad al benznidazol. Asimismo, Luna, *et al.*^10^ y Mejía-Jaramillo, *et al.*^11^, al evaluar cepas pertenecientes a una misma unidad taxonómica discreta (TcI) de diferentes huéspedes y áreas geográficas, observaron diferencias en cuanto al grado de sensibilidad al benznidazol. Teston, *et al.*^45^, por su parte, en pruebas *in vivo* no observaron el predominio de cepas de una determinada unidad taxonómica discreta con un patrón particular de sensibilidad al benznidazol, como tampoco Gruendling, *et al.*^46^, en estudios experimentales en ratones inoculados con *T. cruzi* I, II y IV. Por lo tanto, se considera que las cepas pertenecientes a una misma unidad taxonómica discreta pueden mostrar diferentes rangos de sensibilidad, lo que, al parecer, es una característica inherente a cada cepa.

En general, los clones de Paraguay fueron más sensibles al nifurtimox que al benznidazol y poco sensibles al posaconazol. Estos resultados concuerdan con estudios previos en cepas de Colombia y Venezuela en los que, en ensayos *in vitro,* se encontró que el nifurtimox era más activo que el benznidazol ^10^^,^^33^. Si bien el mecanismo de activación de estos fármacos es parecido, presentan algunas diferencias en sus propiedades farmacológicas que podrían influir en su eficacia en las cepas de *T. cruzi*^47^^,^^48^.

En el presente estudio, los clones evaluados resultaron poco sensibles al posaconazol en comparación con los otros dos fármacos, ya que fue necesario aumentar la concentración de este último para obtener los valores de las concentraciones inhibitorias en el mismo periodo (72 horas) y evidenciar las diferencias entre los clones en cuanto a la sensibilidad al fármaco. En estudios previos de evaluación del efecto del fármaco *in vitro,* se ha reportado que, comparado con los fármacos de referencia, el posaconazol era activo en menores concentraciones, en algunos casos incluso en el rango nanomolar, en diferentes cepas de *T. cruzi,* aunque sin alcanzar la eliminación completa del parásito en cultivos celulares infectados ^49^^-^^51^. Moraes, *et al*. ^49^, consideran al benznidazol y el nifurtimox como compuestos de acción tripanocida rápida y al posaconazol como de acción más lenta, y sugieren que, extendiendo el tiempo de exposición a este último fármaco, se podría alcanzar el 100 % de eficacia.

Los resultados obtenidos con el clon JFcl1 son muy interesantes (altos valores de IC_90_ con el benznidazol), más aún si se tiene en cuenta que la cepa original provenía de un caso clínico humano con reactivación de la infección por uso de inmunosupresores ^52^, aunque no se sabe si el paciente había recibido previamente tratamiento con benznidazol, por lo cual no puede deducirse si esa baja sensibilidad al fármaco es una característica natural del clon o fue inducida por el tratamiento previo. El clon ARcl1 presentó los valores de IC_90_ más altos con el nifurtimox en los clones de Paraguay. Dicho clon correspondía a un caso clínico humano, y no se presumió un contacto previo con este fármaco, ya que en Paraguay el medicamento comúnmente usado es el benznidazol ^53^. Así pues, tal característica sería natural en este clon, lo que también sería el caso del MA194cl1, aislado de un armadillo en el que se registró la más baja sensibilidad al posaconazol.

El clon T589cl1 se destacó por sus altos valores de lC_50_ e lC_90_ con los tres fármacos. El aislamiento original provenía de un triatomino intradomiciliario del Chaco y, considerando que no había habido uso previo de estos fármacos (lo que podría haber inducido la baja sensibilidad observada), esta sería una característica inherente a este clon. En estudios previos tanto *in vitro* como *in vivo,* se ha evidenciado que ciertas cepas de *T. cruzi* presentan resistencia cruzada a diferentes fármacos, por ejemplo, cepas resistentes simultáneamente al benznidazol y al nifurtimox ^9^^,^^10^^,^^36^ o al benznidazol y el posaconazol ^54^^-^^56^.

En el caso de la resistencia cruzada al benznidazol y el nifurtimox, algunos autores han propuesto que podría deberse a que comparten los mecanismos de activación y que las alteraciones en la expresión de las enzimas que participan en estos procesos llevarían a la aparición de esta característica ^34^^,^^36^. En cuanto a la resistencia cruzada al benznidazol y el posaconazol, poco se sabe, ya que los dos fármacos no comparten procesos bioquímicos similares en sus mecanismos de acción y no se han detectado alteraciones en el gen que codifica la enzima lanosterol 14a-dimetilasa (CYP51), principal blanco del antifúngico posaconazol, ni en el cromosoma en el que se ubica este gen en las cepas de *T. cruzi* con resistencia a los dos fármacos ^56^. Estos aspectos deben ser abordados en mayor profundidad en futuros estudios.

Entre los clones de Paraguay estudiados, algunos fueron muy sensibles a un fármaco y menos sensibles a otro, mostrando así un comportamiento diferencial en cuanto a la sensibilidad. En estudios previos, se ha verificado esta resistencia selectiva de las cepas de *T. cruzi* a los medicamentos ^43^^,^^57^^,^^58^, aunque las causas se desconocen. Esto tiene su relevancia en el seguimiento del tratamiento, ya que es posible que el fármaco seleccionado sea más efectivo en algunas cepas de *T. cruzi* y otro lo sea en otras.

La información aquí presentada permite avanzar en el conocimiento de la biología básica de las poblaciones de *T. cruzi* que circulan en Paraguay, especialmente en lo referente a su grado de sensibilidad a los medicamentos utilizados en el tratamiento. Los clones biológicos que mostraron diferente grado de sensibilidad quedan disponibles para futuros estudios en modelo de ratón, en los que se determine la virulencia y la patogenia y se evalúen los otros estadios (amastigotes y tripomastigotes), previa normalización de los índices de infección en cultivos celulares y establecimiento de curvas de crecimiento y tasa de metaciclogénesis, con el fin de estudiar los mecanismos bioquímicos y moleculares de resistencia y sensibilidad a los fármacos y buscar biomarcadores de resistencia natural, aspectos que son relevantes para mejorar el tratamiento y el pronóstico de la enfermedad de Chagas.
